# The Isolation and Characterization of Goose Astrovirus Genotype 2 from Laying Hens with Nephritis in Shandong Province, China

**DOI:** 10.1155/2023/8515116

**Published:** 2023-04-24

**Authors:** Feng Wei, Xiaoning Jiang, Dalin He, Qianqian Wang, Youxiang Diao, Yi Tang

**Affiliations:** ^1^College of Animal Science and Technology, Shandong Agricultural University, 61 Daizong Street, Tai'an, Shandong Province 271018, China; ^2^Shandong Provincial Key Laboratory of Animal Biotechnology and Disease Control and Prevention, Tai'an, Shandong 271018, China; ^3^Shandong Provincial Engineering Technology Research Center of Animal Disease Control and Prevention, Tai'an, Shandong 271018, China

## Abstract

Goose astrovirus genotype 2 (GoAstV2), as a contagious pathogen of fatal visceral gout in goslings, has been widely distributed in major goose-producing regions in China since 2017, leading to significant economic losses to the Chinese goose industry. In this study, a novel goose astrovirus (GoAstV-SDHZ) was isolated and identified from laying hens with nephritis for the first time. Phylogenetic analysis of the sequenced genome of ORF2 gene revealed that the GoAstV-SDHZ strain clustered into Group 2 GoAstVs and shared the highest identity with the other representative GoAstV2 in the nucleotide (ORF1a: 97.2–99.7%; ORF1b: 98.4–99.8%; ORF2: 97.2–99.9%) and amino acid sequence (ORF1a: 98.2–100%; ORF1b: 98.8–99.6%; ORF2: 97.4–99.6%). In summary, this study provides the first evidence of the GoAstV2 infection in Chinese laying hens, which raises potential threat to the poultry industry in China.

## 1. Introduction

Goose astrovirus (GoAstV) is divided into two distinct genotypes (GoAstV1 and GoAstV2) based on the capsid sequences [1]. Its genome is composed of a 5′-untranslated region (UTR), three open reading frames (ORFs), a 3′-UTR, and a poly (A) tail [2]. GoAstV2 was first reported in geese with visceral gout in Shandong province, China in 2017, after which it has quickly spread throughout major goose-breeding provinces in China, including Henan, Hunan, Heilongjiang, Anhui, Shandong, Jiangsu, and Guangdong [3–6]. The mortality rate of the disease was over 30% and resulted in substantial economic losses to the goose industry of China [7–9]. As a novel member of astroviruses, GoAstV2 has a cross-species transmission ability. It not only infects geese but also can cause fatal visceral gout in Cherry Valley ducklings and Muscovy ducklings [10, 11]. The artificial infection experiments confirmed that the GoAstV2 infection in chickens caused similar clinical symptoms to the GoAstV2 infected goslings [1]. In addition, the potential for vertical transmission of GoAstV2 poses a great challenge to the prevention and control of the disease [12]. However, natural infection of GoAstV2 in laying hens has not been identified in China so far. Here, we isolated and identified a goose astrovirus genotype 2 from laying hens with nephritis for the first time.

## 2. Materials and Methods

### 2.1. Ethics Statement

This study was approved by the Animal Care and Use Committee of Shandong Agricultural University (permit number: 20190322) and performed in accordance with the ““Guidelines for Experimental Animals”” of the Ministry of Science and Technology (Beijing, China).

### 2.2. Sample Collection

In May 2022, tissue samples (kidneys) from diseased laying hens (Hainan brown laying hens farm) exhibiting typical symptoms of nephritis were collected by our team. The Hainan brown laying hens farm (>20000 birds) was located in the southern part of Shandong Province, China. The diseased laying hens were 300-day-old. The tissue samples from diseased laying hens were collected for virus detection and identification.

### 2.3. Pathogen Screening

Viral genome was extracted from kidneys using a TransGen Virus DNA/RNA kit (TransGen Biotech, Beijing, China) according to the manufacturer's instructions. The routine surveillance of laying hen pathogens, including avian nephritis virus (ANV), chicken astrovirus (CAstV), Tembusu virus (TMUV), fowl adenovirus (FAV), avian influenza virus (AIV), infectious bronchitis virus (IBV), Newcastle disease virus (NDV), GoAstV1, and GoAstV2 were examined by RT-PCR or regular PCR using a one-step RT-PCR kit (TransGen Biotech, Beijing, China). The specific primer sequences are listed in Table 1.

### 2.4. Virus Isolation and Genome Sequencing

The supernatants of GoAstV2-positive tissue samples were filtered by passing through 0.22 *μ*m filter for virus isolation. Supernatants were inoculated into Leghorn chicken hepatocellular carcinoma (LMH, ATCC) cells and cultured at 37°C with 5% CO_2_. After three consecutive passages, the positive cell cultures were harvested for the detection of GoAstV2 and then preserved at −80°C for further sequencing. Nucleotide acid was extracted from the positive cell-cultures, and the whole-genome sequencing of the positive cell-culture isolates was investigated by PCR assay with the previously reported primer sets [4]. PCR and sequencing analysis were performed at least 3 times.

### 2.5. Phylogenetic Analysis and Sequence Alignments

The identities of the nucleotide and deduced amino acid sequences were aligned using the ClustalW software (MegAlign). Phylogenetic trees of the three open reading frames (ORFs 1a, 1b, and 2) were conducted by the maximum-likelihood method implemented in MEGA 6.0 software with 1000 bootstrap replications [13]. Moreover, the sequence alignments of ORF2 amino acid sequences were implemented using DNAMAN software and visualized by colored diagrams.

### 2.6. Histopathology Examination

Tissues (e.g., kidney) collected from the diseased laying hens were fixed with a 10% neutral buffered formalin. The tissue was sliced into five-micron slices, stained using standard methods, and then observed under a light microscopy (Nikon, EclipseE100, Japan).

## 3. Results

**Figure 1:**
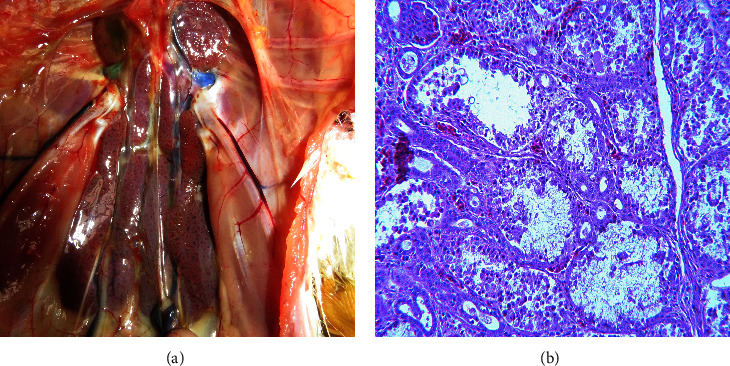
Pathological and histopathological lesions in laying hens. (a) Severe nephritis of swollen and haemorrhage of kidneys and urate crystals depositing in ureters. (b) Degeneration, necrosis, and exfoliation of renal tubular epithelial cells (200x).

In May 2022, a Hainan brown laying hens farm raising 300 day-old laying hens (>20000 birds) in Shandong Province, China, reported an outbreak of diarrheal disease. At autopsy, the kidneys were enlarged with slight urate deposition in the ureter (Figure 1(a)) and no significant changes in the follicles. Furthermore, the main histopathological changes of the kidney revealed degeneration, necrosis, and exfoliation of renal tubular epithelial cells (Figure 1(b)).

**Figure 2:**
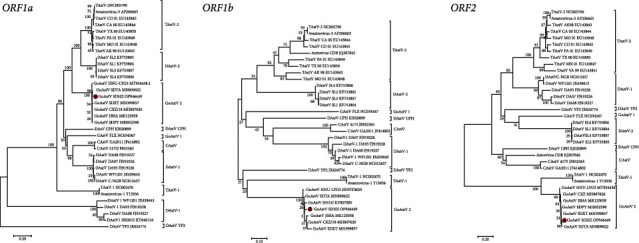
Phylogenetic relationship analysis based on nucleotide sequences of ORF1a, ORF1b, and ORF2 of the GoAstV-SDHZ strain (•) and other AstVs. The trees were generated using MEGA 6.0 software and the neighbor-joining method with 1000 bootstrap replicates. The GoAstV-SDHZ isolate determined in this work is indicated by a red dot.

Five kidney samples (5/8) were identified to be GoAstV2 positive by RT-PCR using the above primers, and the common poultry-origin viruses were negative. After three consecutive passages, one GoAstV2 strain designated as GoAstV-SDHZ was successfully isolated from the samples using LMH cells. The complete genome of GoAstV-SDHZ strain was obtained and submitted to the GenBank (Accession numbers: OP946449). The full-length genome sequence of GoAstV-SDHZ strain was 7,166 nt in length, with a 5′-UTR of 13 nt, 3 overlapping ORFs, and a 3′-UTR of 224 nt. ORF1a of the isolate was 3,255 nt (positions 14 to 3268) in length, encoding a 1085 amino acids nonstructural polyprotein. The predicted ORF1b gene of the isolate was 1,551 nt (positions 3259 to 4809) in size, encoding the RNA dependent RNA polymerase (RdRp). The predicted ORF2 gene of the isolate was 2115 nt (positions 4828 to 6942) in size, encoding a 705 amino acids capsid protein. To further investigate the evolutionary relationships between GoAstV-SDHZ and other representative avian members, a phylogenetic tree was constructed based on the deduced nucleotide sequences of the ORF1a, ORF1b, and ORF2 homologous genes with MEGA 6.0 software. As shown in Figure 2, all the three trees demonstrated that the GoAstV-SDHZ strain was clustered together with the GoAstV2 strains.

The deduced amino acid sequences of the three ORFs of the GoAstV-SDHZ strain were compared with other representative avian members. As described in Table 2, the GoAstV-SDHZ strain shared the highly similar, with the GoAstV2 strain, in terms of the genome sequence (ORF1a: 97.2–99.7%; ORF1b: 98.4–99.8%; ORF2: 97.2–99.9%) and amino acid sequence (ORF1a: 98.2–100%; ORF1b: 98.8–99.6%; ORF2: 97.4–99.6%).

**Figure 3:**
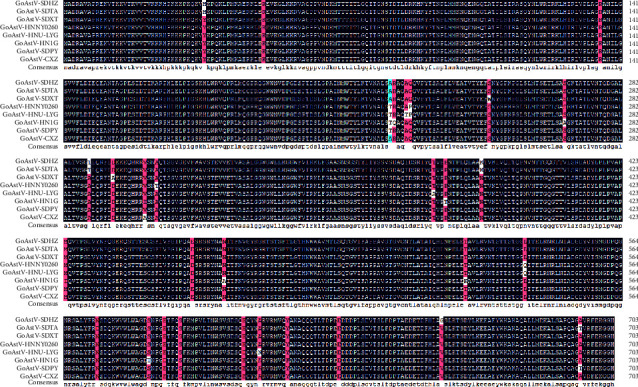
Visual analysis of amino acid sequences of GoAstV2 capsid proteins.

Moreover, the visual analysis of ORF2 amino acid sequences alignment of GoAstV2 strains indicated that the capsid protein contained the most mutations, including a unique mutation (V660 G) possessed by GoAstV-SDHZ strain (Figure 3).

## 4. Discussion

Since 2017, GoAstV2 associated gout has caused huge economic damage to the waterfowl industry [4, 14]. In this study, a novel goose astrovirus (GoAstV-SDHZ) has been isolated and identified from laying hens with nephritis. To the best of our knowledge, this is the first report of natural infection of goose astrovirus genotype 2 in laying hens, indicating that a widespread tendency and cross-host transmission from waterfowl to domestic chicken has occurred. Astroviruses are transmitted horizontally, mainly by the fecal-oral route [15, 16]. Liu et al. confirmed the horizontal and vertical transmission of duck astrovirus CPH by testing fresh duck manure and embryos from hatcheries in different provinces of China [17]. GoAstV2 has been found to transmit vertically from breeding geese to goslings, and leads to reduced fertilization and hatchability of embryos [12]. Based on the above reports, we conclude that GoAstV2 may also be transmitted vertically, but whether the virus infection has an effect on the fertilization rate and hatching rate of laying hens needs further research to clarify these issues. The GoAstV-SDHZ strain shared the highest identity with the duck-origin GoAstV-SDTA strain in the nucleotide (ORF1a: 99.7%; ORF1b: 99.8%; ORF2: 99.9%) and amino acid sequence (ORF1a: 100%; ORF1b: 99.6%; ORF2: 99.6%). This may be due to improper disinfection of feed transport vehicles, or the failure of breeders to disinfect themselves after being exposed to infectious sources outside the farm, which eventually led to cross-species transmission from ducks to laying hens. From field cases, there was severe swelling in the kidneys and no other gout symptoms were found, which were different from the symptoms reported previously in waterfowl [3, 18]. The exact mechanism of cross-species transmission and differences in virulence of astrovirus remains unclear and needs further research to confirm.

## 5. Conclusions

Taken together, this study provides the first evidence of goose astrovirus genotype 2 natural infection in Chinese laying hens, which raises potential threat to the poultry industry in China. For this reason, further studies are needed to explore the pathogenicity of GoAstV-SDHZ strain in laying hens.

## Figures and Tables

**Table 1:** Primers used in this study for detection of the viruses.

Primer name	Sequence (5′ ⟶ 3′)	Product size (bp)
ANV-F	CCAGGAGACCAACAAGA	453
ANV-R	CGCATAGCACGCATT	
CAstV-F	TGAACAATGAGCCAGAG	427
CAstV-R	CCACCAAAGAAGGGA	
TMUV-F	GCCACGGAATTAGCGGTTGT	401
TMUV-R	TAATCCTCCATCTCAGCGGTGTAG	
H9-AIV-F	GATAGAGACTCAACCCAAAA	315
H9-AIV-R	AACATCCTTTCCCATCTTCC	
IBV-F	AACTGAACAAAAGACAGACTT	1711
IBV-R	AACATAAGGGCAATTTGCA	
FAV-F	TGGACATGGGGGCGACCTA	1219
FAV-R	AAGGGATTGACGTTGTCCA	
GoAstV1-F	TGGTGCGAAAGGAGG	564
GoAstV1-R	GGTTGACATAGCATAGCG	
GoAstV2-F	ATTCTTGGCTCGGTTGTC	489
GoAstV-R	CCTGTGTTGCTCCTTCTC	
NDV-F	TCCCRAATATACCCAKRGAT	1038
NDV-R	GTTTTGCGATATGATACCAGGAG	

**Table 2:** Sequence identities between GoAstV-SDHZ strain and selected representative astroviruses.

		Sequence identity (%)					
		ORF 1a		ORF 1b		ORF 2	
Species	Virus strain	nt	aa	nt	aa	nt	aa
GoAstV	SDTA	99.7	100	99.8	99.6	99.9	99.6
	SDXT	97.8	98.2	98.8	98.8	99.1	98.9
	HNU-LYG3	98.2	98.7	98.7	99.6	98.2	98.2
	HNNY0260	98.4	99.2	98.4	99.4	98.6	98.6
	CXZ	97.5	99.2	99.0	99.6	99.2	99.0
	HN1G	97.2	98.8	99.1	99.0	97.2	97.4
	SDPY	97.8	99.3	99.2	99.6	99.1	99.0

DAstV	DA08	53.5	47.7	65.8	64.1	57.9	56.0
	YP2	51.1	41.8	63.6	61.4	48.0	34.3
	SL1	61.5	58.2	68.8	68.1	47.1	38.0

TAstV	PA01TA1	62.2	59.2	68.8	68.9	60.9	56.6
	TA1	49.7	39.5	60.2	60.7	47.9	41.3

CAstV	GA2011	55.6	48.5	65.3	65.2	48.4	37.2

## Data Availability

The data and materials that support the findings of this study are available from the corresponding author, Youxiang Diao: yxdiao@126.com, upon reasonable request.
